# Editorial of Special Issue “Diet and Nutrition during Chemotherapy and Radiotherapy”

**DOI:** 10.3390/nu14122422

**Published:** 2022-06-10

**Authors:** Peter Meade Anderson

**Affiliations:** Departments of Pediatric Hematology/Oncology and Bone Marrow Transplant, Cleveland Clinic Pediatric and Taussig Cancer Institutes, R3 9500 Euclid, Cleveland, OH 44195, USA; andersp@ccf.org; Tel.: +1-216-308-2706

Diet and nutrition during chemotherapy and radiotherapy can be quite challenging for the cancer patient and their caregivers. Therefore, this Special Issue provides new information and additional resources for patients, families, nutrition professionals, and oncologists. Nutrient intake during head and neck chemo-radiotherapy can be particularly challenging, not just during therapy [1,2] but after its completion, as shown by Abu Zaid et al. [3]. Fanetti et al., provide evidence that prognostic nutritional index predicted >10% weight loss and late mucositis, being significantly associated with a worse overall survival [4]. Lin et al., compared concurrent chemoradiotherapy for advanced head and neck carcinoma involving the oral cavity and non-oral cavity [1]. This study showed how different factors independently correlated with interval changes in body composition parameters, particularly for lean body mass, total fat mass, and absolute lymphocyte count (ALC). Their observations highlighted mechanisms by which nutrition could affect the prognosis of patients those with head and neck squamous cell carcinoma.

Lee et al., investigated the feasibility of using patient-reported outcomes (PRO) to predict changes in body composition in women with gynecologic cancer undergoing post-operative pelvic radiotherapy. This study showed a highly significant (*p* < 0.001) association of PRO and an increased risk of muscle loss [5]. Since sarcoopenia is associated with a worse survival in some cancers [2], this study may provide a justification and tool for more timely interventions. One such intervention, oral nutritional supplements, improved body composition and prevented hypoalbuminemia in women with breast cancer, as explored by Grupinska et al. [6]. Antioxidant capacity may also have a protective effect on women receiving adjuvant treatment for breast cancer, as demonstrated in the biomarker study by Reitz et al. [7]. Other interventions, particularly regarding the effects of nutrition by amelioration of mucositis, esophagitis, and enteritis, were reviewed by Garcia-Guzalbo et al. [8] and studied in detail by Anderson et al. [2]. The latter review includes some practical approaches that anticipate and ameliorate chemotherapy and radiation toxicities that affect eating. This review also provides information about the many strategies available for improving nutrition in order to reduce toxicity. These include (a) reducing nausea and vomiting, (b) decreasing mucosal damage, (c) avoiding sarcopenia, and (d) developing therapeutic healthy relationships between patients, caregivers, and oncology professionals [2]. In a cross-sectional study, Poulia et al., compared changes in nutritional parameters during an initial evaluation with changes on quality of life, progression-free survival, and overall survival in pancreatic cancer [9]. Although insulin-like growth factors and binding proteins were not significantly different after hemaopoietic stem cell transplant compared to controls [10], the search for predictive biomarkers with better nutritional outcomes after chemotherapy and/or radiation continues.

This Special Issue includes research on the modification of gut microbiota associated with radiation, which is another variable related to diet and nutrition that may affect cancer survival [2,11]. Belnager et al. showed that early nutritional interventions in pediatric oncology patients were feasible [12]. Although short-term calorie reduction was associated with benefits for the post chemotherapy parameters of lymphoma patients, including lymphocyte counts as studied by Tang et al. [13], intermittent fasting using 12–15 h instead of 48 h time periods may be more practical for a family-centered care approach [2]. Overall, the 13 papers included in this Special Issue highlight numerous state-of the art scientific contributions and the importance of diet and nutrition during chemotherapy and radiation. This issue also presents current efforts to improve nutrient intake and quality of life, both during and after chemotherapy and radiotherapy.
